# Delayed Cerebral Radiation Necrosis after Neutron Beam Radiation of a Parotid Adenocarcinoma: A Case Report and Review of the Literature

**DOI:** 10.1155/2014/717984

**Published:** 2014-09-30

**Authors:** Christopher S. Hong, Hamza N. Gokozan, José J. Otero, Michael Guiou, J. Bradley Elder

**Affiliations:** ^1^Department of Neurological Surgery, The Ohio State University Wexner Medical Center, 410 W. 10th Avenue, Doan Hall N1052, Columbus, OH 43210, USA; ^2^Department of Pathology, The Ohio State University Wexner Medical Center, Columbus, OH 43210, USA; ^3^Department of Radiation Oncology, The Ohio State University Wexner Medical Center, Columbus, OH 43210, USA

## Abstract

Cerebral radiation necrosis (CRN) is a well described possible complication of radiation for treatment of intracranial pathology. However, CRN as sequelae of radiation to extracranial sites is rare. Neutron beam radiation is a highly potent form of radiotherapy that may be used to treat malignant tumors of the salivary glands. This report describes a patient who underwent neutron beam radiation for a parotid adenocarcinoma and who developed biopsy-confirmed temporal lobe radiation necrosis thirty months later. This represents the longest time interval described to date, from initial neutron radiation for extracranial pathology to development of CRN. Two other detailed case studies exist in the literature and are described in this report. These reports as well as our patient's case are reviewed, and additional recommendations are made to minimize the development of CRN after extracranial neutron beam radiation. Physicians should include the possible diagnosis of CRN in any patient with new neurologic signs or symptoms and a history of head and neck radiation that included planned fields extending to the base of the skull. Counseling of patients prior to neutron beam radiation should include potential neurologic complications associated with CRN and risks of treatment for CRN including neurosurgical intervention.

## 1. Introduction

Cerebral radiation necrosis (CRN) is a delayed phase reaction of normal brain tissue that typically occurs within six months after cranial radiation. The resulting symptoms depend on the size, anatomic location, and extent of associated edema. Common presentations include motor/sensory deficits, cognitive dysfunction, headaches, and new onset seizures [1]. Tissue damage from CRN is generally considered irreversible and, if untreated, can incur permanent deficits or death [2, 3]. Medical management with corticosteroid therapy can lead to resolution of the CRN and associated symptoms. However, large areas of CRN may not respond to medical management and lead to progressive symptoms in a similar fashion to intrinsic brain tumors. In these cases, surgical intervention for removal of the CRN may be required. CRN has been well described in the literature as a potential complication after radiation therapy (RT) for primary and metastatic brain tumors. There is an approximate 5% risk for CRN after cranial radiation with conventional fractionation at doses of 55–60 Gy [4]. Even higher incidences of CRN have been reported after stereotactic radiosurgery (SRS) for primary or metastatic brain tumors, with 2-year post-SRS CRN rates between 11% and 50% [5–7]. Although CRN has been reported as late as 47 years after RT for a pilocytic astrocytoma, the vast majority of cases after radiation of an intracranial lesion occur between 6 and 12 months [3, 8].

Conversely, CRN after radiation of extracranial neoplasms is much less common and less well described. These cases typically occur as sequelae of radiation for tumors of the head and neck, most commonly nasopharyngeal carcinoma (NPC), and involve conventional forms of RT like photons, gamma rays, and protons [11–14]. Neutron beam radiation is a highly potent form of radiotherapy that inflicts greater biologic damage than the equivalent dose of X-rays. An often cited measure reflecting these differences is relative biologic effectiveness (RBE), defined as D250/Dr, where D250 and Dr are the doses of X-rays and test radiation required for equivalent biologic effect. Neutrons inflict damage via direct interaction with a cell's DNA (versus the highly modifiable indirect interaction of X-rays) or strike other elements (e.g., carbon, oxygen) to produce highly damaging spallation products (alpha particles) and are less dependent upon oxygen to produce cell death as compared to X-rays. Studies have demonstrated the efficacy of neutron beam radiation against certain radioresistant tumors, including soft tissue and cartilaginous sarcomas, prostate cancer, non-small cell lung cancer, and head and neck cancers [15–19]. In normal tissue, the RBE of neutron radiation at clinically relevant fractionated dosing (200 rad/fraction) is 2.2 in lungs, 2.9 in skin, 3.8 in intestine, and as high as 4.5 to 5.2 in the central nervous system [20–23]. Due to an abundance of hydrogen atoms by virtue of high lipid content, the brain is thought to be particularly susceptible to neutron radiation-induced damage via formation of free radicals and oxidative stress [24].

CRN as sequelae of neutron beam radiation for salivary gland tumors has been mentioned in prior retrospective studies but not described in sufficient detail to allow counseling of patients currently treated with this algorithm [18, 25, 26]. We present a case of a patient who developed CRN of the temporal lobe 30 months after neutron beam radiation for recurrent parotid adenocarcinoma. This is the first report of CRN after neutron beam radiation to an extracranial site since 1992. The present patient also represents the longest delay from neutron beam radiation for an extracranial tumor to the development of CRN. Thus, continued monitoring for symptoms and neurologic complications associated with CRN must continue for at least this time frame in patients who receive neutron beam radiation for extracranial tumors.

## 2. Case Report

A 68-year-old woman began to have symptoms of mild pain and short episodes of otalgia over the left side of her face. Over the next two years, her symptoms worsened, and she was presumed to have Ramsey-Hunt syndrome. Because the patient did not experience any improvement with antiviral treatment, an MRI of her left face was obtained, which showed a 1.7 cm mass in the deep lobe of the left parotid gland. A positron emission tomography (PET) scan was performed, which demonstrated increased uptake with a maximum standardized uptake value (SUV) of 4.8 and a left lower lobe lung nodule measuring 1.5 cm. These findings likely indicated advanced malignancy of the parotid gland, for which the patient underwent a left total parotidectomy, supraomohyoid neck dissection, and mastoidectomy. The final pathology report confirmed acinar cell carcinoma of the left parotid gland. Biopsies of eleven total lymph nodes as well as the left facial nerve were negative for disease. A few months after surgery, she underwent a left exploratory thoracotomy with excisional biopsy of the lung nodule. Pathology confirmed metastatic carcinoma with absent mediastinal and hilar lymph node involvement.

**Figure 1:**
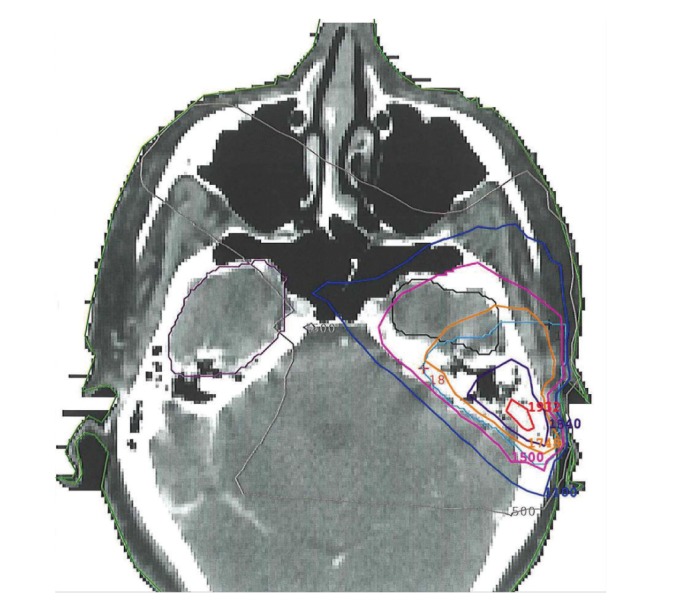
A representative axial slice from the patient's radiation plan is shown. The left temporal lobe of the brain is contoured in black. This area received 1678 centi-nGy and falls between 1500 centi-nGy (pink) and 1748 centi-nGy (orange) isodose lines. The treatment isocenter received a total dose of 1819 centi-nGy.

**Figure 2:**
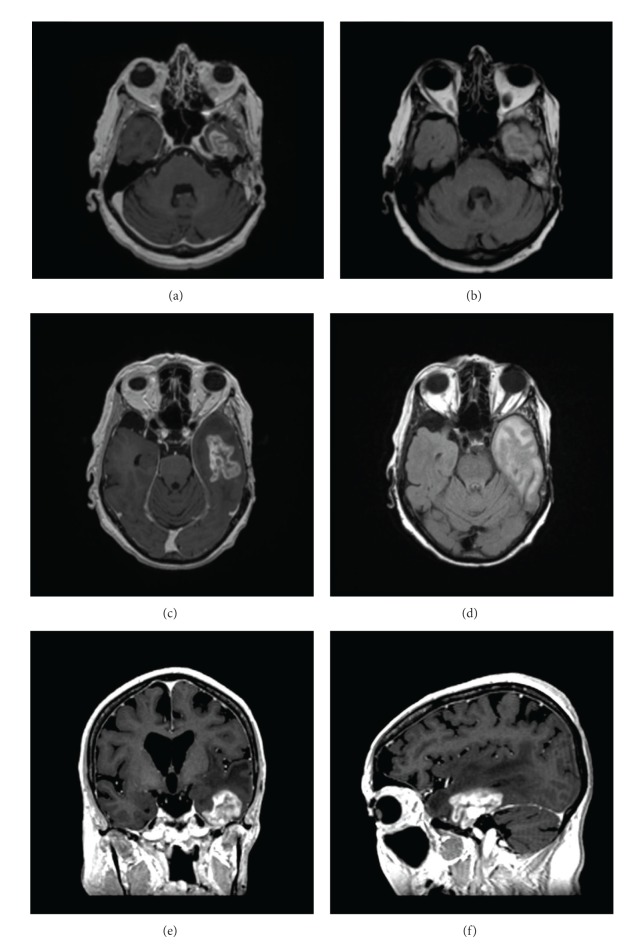
Axial slices of T1 postcontrast (a) and T2 FLAIR (b) MRIs are shown, corresponding to the radiation plan seen in Figure 1. Additional axial T1 postcontrast (c), axial T2 FLAIR (d), coronal T1 postcontrast (e), and sagittal T1 postcontrast (f) slices depict an irregularly enhancing mass lesion in its greatest dimensions, involving the left temporal lobe tip. The lesion measured 4.6 cm in the greatest dimension with significant surrounding edema. There was evidence of temporal bone resection, consistent with prior resection of the left parotid gland.

**Figure 3:**
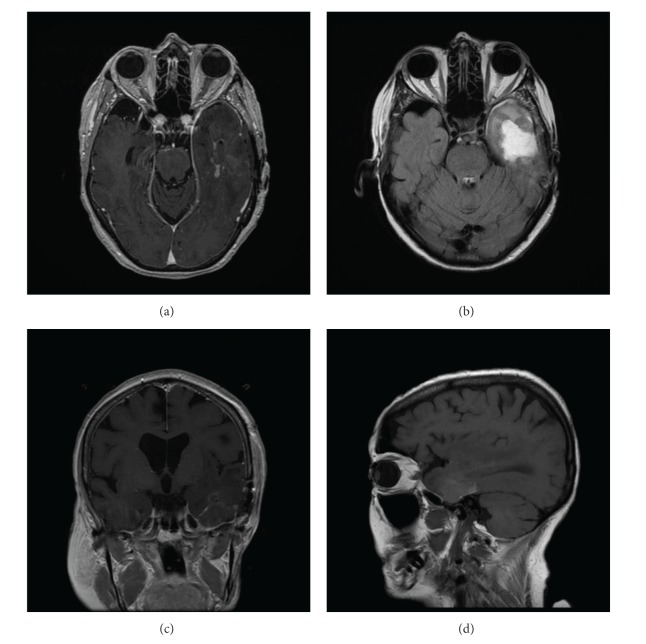
Axial T1 postcontrast (a), T2 FLAIR (b), coronal T1 postcontrast (c), and sagittal T1 postcontrast (d) MRIs obtained on postoperative day 1 demonstrated total resection of the enhancing mass seen on preoperative imaging. There were some minor blood products and enhancement, consistent with expected normal reactive change after surgery.

**Figure 4:**
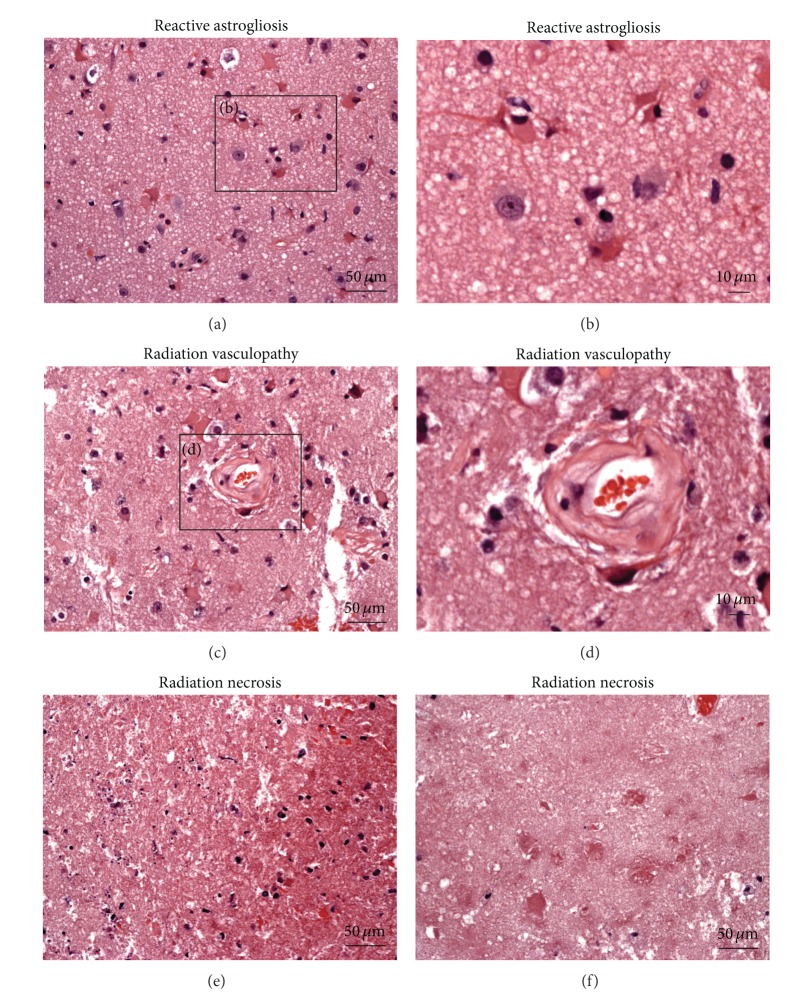
Formalin fixed, paraffin embedded tissue sections stained with hematoxylin and eosin demonstrate reactive astrogliosis, radiation-induced vasculopathy, and necrosis. (a) Reactive astrocytes show hypertrophic, eosinophilic cytoplasm with eccentrically placed nuclei. (b) Higher magnification of inset in (a). (c) Hyalinized arterioles are seen, characteristic of radiation-induced vasculopathy. (d) Higher magnification image of (c). Radiation necrosis is demonstrated at (e) interface of brain and necrotic area and (f) necrosis in the center of the lesion.

Given the presence of locally invasive disease, the patient was referred to radiation oncology for consideration of adjuvant neutron beam radiation. She received 16 fractions of 1.15 neutron nGy/fraction over four weeks for a total dose of 18.4 nGy (Figure 1). Adverse events after radiation were limited to grade 2 mucositis and grade 1 erythema of the skin in the treatment field. Twenty months after completion of radiotherapy, the patient developed temporal bone necrosis, which was successfully resected by otolaryngology. At this time, brain imaging did not demonstrate any focal parenchymal involvement. Thirty months after neutron beam radiation, the patient developed progressive dysnomia, memory impairment, expressive aphasia, and personality changes. An MRI revealed a heterogeneous, irregular, ring-enhancing lesion in the left temporal lobe, concerning for tumor metastasis. However, radiation necrosis was also considered part of the differential diagnosis given there was enhancement in an area corresponding to the original radiation plan (Figure 2). She was initially treated with high dose dexamethasone, with some improvement in her symptoms. However, given her continued symptoms, steroid dependence, and surgical accessibility of the lesion, surgical resection was recommended. A left temporal craniotomy was performed with gross total resection of the lesion (Figure 3). There was no erosion of the floor of the middle fossa or overlying dura or other evidence of direct communication from the lesion to any extracranial compartment. Final pathology demonstrated reactive astrogliosis and hyalinized arterioles, characteristic of radiation necrosis, with no evidence of tumor (Figure 4). Within one month of surgery, her neurologic status had returned to baseline with full recovery of speech and memory. Follow-up imaging revealed no evidence of recurrence of radiation necrosis. Steroids were tapered off within four weeks of surgery. At two-year followup, she was doing well with no evidence of tumor, CRN, or neurologic dysfunction.

## 3. Discussion

Currently, adjuvant therapy for malignant parotid tumors may include neutron beam radiation, which in some reports has demonstrated better outcomes compared to conventional photon radiotherapy [18, 19, 27]. The first studies seeking to demonstrate the safety and efficacy of neutron beam radiation had higher incidences of severe late toxicities, on the order of 8.9%–17%, including a few of cases of CRN. Unfortunately, most of these reports do not provide further clinical details beyond radiation dosing. In a retrospective analysis of 279 patients with salivary gland cancers treated with neutron beam radiation at the University of Washington Neutron Facility, four developed what the authors described as “central nervous system radiation necrosis” [18]. Further details regarding tumor type, prior treatment, location of CRN, clinical management of the CRN, and overall survival were not provided. These 4 patients, like the other 275 in this study, had received a total dose between 17.4 and 20.7 nGy to their primary tumor site before developing CRN. Two cases of CRN have also been reported in patients undergoing treatment at Fermilab at Northern Illinois University between 1976 and 1984 [25]. These patients, however, received relatively high total doses, ranging from 20 to 28 nGy. Three reports of CRN were included in a retrospective study of patients treated at iThemba Laboratory for Accelerator Based Science (LABS) in Cape Town, South Africa, all of whom received neutron beam radiation prior to 1992 [28]. Notably, the authors stated that CNS morbidity did not occur when brain doses were under 13 nGy. Our patient received a total dose of 16.8 nGy to the tip of the left temporal lobe, precisely where her CRN eventually occurred.

Our review of the literature found two other detailed reports of CRN from neutron beam radiation to an extracranial site (Table 1). Diengdoh and Booth described a 47-year-old male with a history of lung adenocarcinoma who presented with symptoms of mild right hemiparesis, gustatory hallucinations, papilledema, and headache [9]. He had undergone neutron beam radiation of unknown total dose to his left parotid gland for treatment of a biopsy-confirmed metastatic lesion. 21 months after receiving radiation, the patient presented with the neurologic symptoms described above and a brain MRI demonstrated a 6 cm contrast-enhancing lesion in the left temporal lobe. This lesion was subsequently resected and found to be necrotic tissue. The patient recovered uneventfully and was asymptomatic seven months after surgery. Manz et al. described a 42-year-old male with a history of recurrent adenocystic carcinoma of the right submandibular gland [10]. He had undergone adjuvant radiation with a calculated tumor dose of 2.080 neutron plus gamma rads to the skull base in 26 fractions. Ten months after radiation, he developed rapidly progressive left-sided hemiparesis and multiple right-sided cranial nerve palsies. Despite medical management, the patient deteriorated, and CRN of the brainstem was diagnosed on autopsy. Our case report, therefore, describes the longest latency period between time of initial neutron beam radiation to an extracranial site and onset of neurological symptoms attributable to CRN.

Photon radiation for salivary gland tumors has been shown to result in CRN, as early as four months after radiotherapy [29]. The vast majority of these patients presented with neurological symptoms within two years of treatment [29–31]. Taken together with the aforementioned studies, our findings suggest that patients undergoing external beam radiation for head and neck malignancies should be followed for at least three years to adequately monitor treatment-related brain toxicity. Patients who received radiation to the parotid region may present with subtle cognitive deficits rather than gross motor and sensory findings. This clinical presentation combined with an enhancing lesion on MRI in the tip of the temporal lobe may be highly suggestive of CRN. Serial surveillance with brain MRI every six months for at least three years may detect early stage CRN. Early detection may allow for complete therapeutic response to medical treatments such as corticosteroids, anticoagulant and antiplatelet therapies, hyperbaric oxygen therapy, and bevacizumab and potentially avoid the need for neurosurgical intervention [7, 32–36].

Base of skull involvement is considered a negative prognostic indicator in the management of salivary gland tumors and portends poor locoregional disease control as well as overall survival [18]. This has been partly attributed to an inability to treat disease above the lower temporal lobes with therapeutic radiation dosing without increased risk of CRN. Previous studies of neutron radiation toxicities have cited 12 nGy as the cut-off value for which CRN can be avoided [26, 37]. Douglas et al. postulated that this dosing limitation leaves disease above the lower temporal lobes, underdosed, and contributes to the decreased progression free survival associated with base of skull involvement [37]. As such, they demonstrated prospectively that a 12 Gy gamma knife boost to the skull base resulted in significantly higher rates of local control after 40 months compared to historical controls (82% versus 39%). However, three of 34 patients treated with gamma knife boost developed evidence of CRN on MR imaging, of whom two remained asymptomatic. The third patient experienced headaches, which was successfully treated with several months of low-dose steroids. In comparison, in the present study, our patient received a dose of 16.78 nGy to the temporal lobe tip, well above the recommendations for 12 Gy or less to this area. Although a few studies have demonstrated that single modality radiation treatment with gamma knife radiosurgery may adequately treat salivary gland tumors, CRN has been reported in these patients as well [38–41]. Furthermore, in a recent retrospective study of 184 head and neck cancer patients treated with gamma knife radiosurgery, approximately 7% of all treated lesions still resulted in CRN to the temporal lobe [42]. The biologic responses of tissue to radiosurgery remain relatively uncharacterized, which further complicates accurate risk assessment of CRN after neutron beam radiation versus radiosurgery. As such, further reports are needed to elucidate whether one form of radiation at therapeutic doses confers greater risk for CRN over another, as well as whether a maximum dose of 12 nGy to the temporal tip circumvents delayed CRN.

## 4. Conclusions

The case report described here illustrates that CRN must be considered as a potential toxicity of neutron radiation for malignant salivary gland tumors. The current literature suggests that total radiation dosing to the temporal lobe should not exceed 12 nGy. In cases of skull base involvement where underdosing to areas above the lower temporal lobes is a concern, gamma knife boost can be considered though this does not totally eliminate the risk of CRN. Alternatively, patients may be observed and administered salvage gamma knife radiosurgery for locally recurrent disease. Furthermore, counseling of patients prior to neutron beam radiation should include potential neurologic complications associated with CRN and risks of treatment for CRN including neurosurgical intervention. Further studies may help elucidate additional risk factors for development of CRN after extracranial radiation, more accurately predict the time course of CRN, and identify patients at higher risk for this complication.

## Figures and Tables

**Table 1:** Prior reports of cerebral radiation necrosis after neutron beam radiation to a salivary gland site.

Author	Patient age/gender	Tumor histology, location	Total radiation dose (nGy)	Presenting neurological symptoms	Latency period until symptomatic cerebral radiation necrosis
Diengdoh and Booth [9]	47/m	Metastasis (lung adenocarcinoma) of parotid gland	Not reported	Hemiparesis dysnomia; headache	21

Manz et al. [10]	42/m	Adenocystic carcinoma of submandibular gland	2.08	Cranial palsies; hemiparesis	10

Index patient	68/f	Acinar cell carcinoma of parotid gland	18.4	Dysnomia, memory loss, aphasia, personality changes	30
